# Impact of Telemedicine Modality on Quality Metrics in Diverse Settings: Implementation Science–Informed Retrospective Cohort Study

**DOI:** 10.2196/47670

**Published:** 2023-07-26

**Authors:** Danielle Rome, Alyssa Sales, Talea Cornelius, Sujata Malhotra, Jessica Singer, Siqin Ye, Nathalie Moise

**Affiliations:** 1 Department of Medicine Columbia University Irving Medical Center New York, NY United States; 2 Columbia University New York, NY United States; 3 Center for Behavioral Cardiovascular Health Department of Medicine Columbia University Irving Medical Center New York, NY United States

**Keywords:** telemedicine, telehealth, implementation science, quality metrics, screening, adoption, diverse, socioeconomic, audio based, video based, video consultation

## Abstract

**Background:**

Video-based telemedicine (vs audio only) is less frequently used in diverse, low socioeconomic status settings. Few prior studies have evaluated the impact of telemedicine modality (ie, video vs audio-only visits) on clinical quality metrics.

**Objective:**

The aim of this study was to assess telemedicine uptake and impact of visit modality (in-person vs video and phone visits) on primary care quality metrics in diverse, low socioeconomic status settings through an implementation science lens.

**Methods:**

Informed by the RE-AIM (Reach, Effectiveness, Adoption, Implementation, and Maintenance) framework, we evaluated telemedicine uptake, assessed targeted primary care quality metrics by visit modality, and described provider-level qualitative feedback on barriers and facilitators to telemedicine implementation.

**Results:**

We found marginally better quality metrics (ie, blood pressure and depression screening) for in-person care versus video and phone visits; de-adoption of telemedicine was marked within 2 years in our population.

**Conclusions:**

Following the widespread implementation of telemedicine during the COVID-19 pandemic, the impact of visit modality on quality outcomes, provider and patient preferences, as well as technological barriers in historically marginalized settings should be considered.

## Introduction

Following initial COVID-19 waves, the reintroduction of in-person care alongside telemedicine provided an opportunity to elucidate nuances in telemedicine implementation and assess its impact compared to in-person visits [1]. Moreover, telemedicine expansion beyond the pandemic warrants evaluation of differences by modalities (ie, video-based vs audio-only telemedicine) [2,3].

Research suggests that the addition of telemedicine (vs in-person care alone) may improve quality measures and clinical outcomes [4-7]. However, findings are inconsistent [8,9] and often exclude or cannot differentiate video from audio-only telemedicine, which is more frequently used in diverse, low socioeconomic status (SES) settings where digital literacy and access issues are known [10-13]. Although video visits (vs audio-only) may impact provider-level outcomes (ie, increased visit duration, number of diagnoses addressed, rates of medication, lab, and imaging orders) [14,15], few prior studies have examined the impact of video and audio-only versus in-person visits on quality metrics in low SES settings.

To address these gaps, we used an implementation science framework and mixed methods approach to assess telemedicine uptake and the impact of modalities on primary care quality metrics in diverse, low SES settings.

## Methods

### Study Population

**Figure 1:**
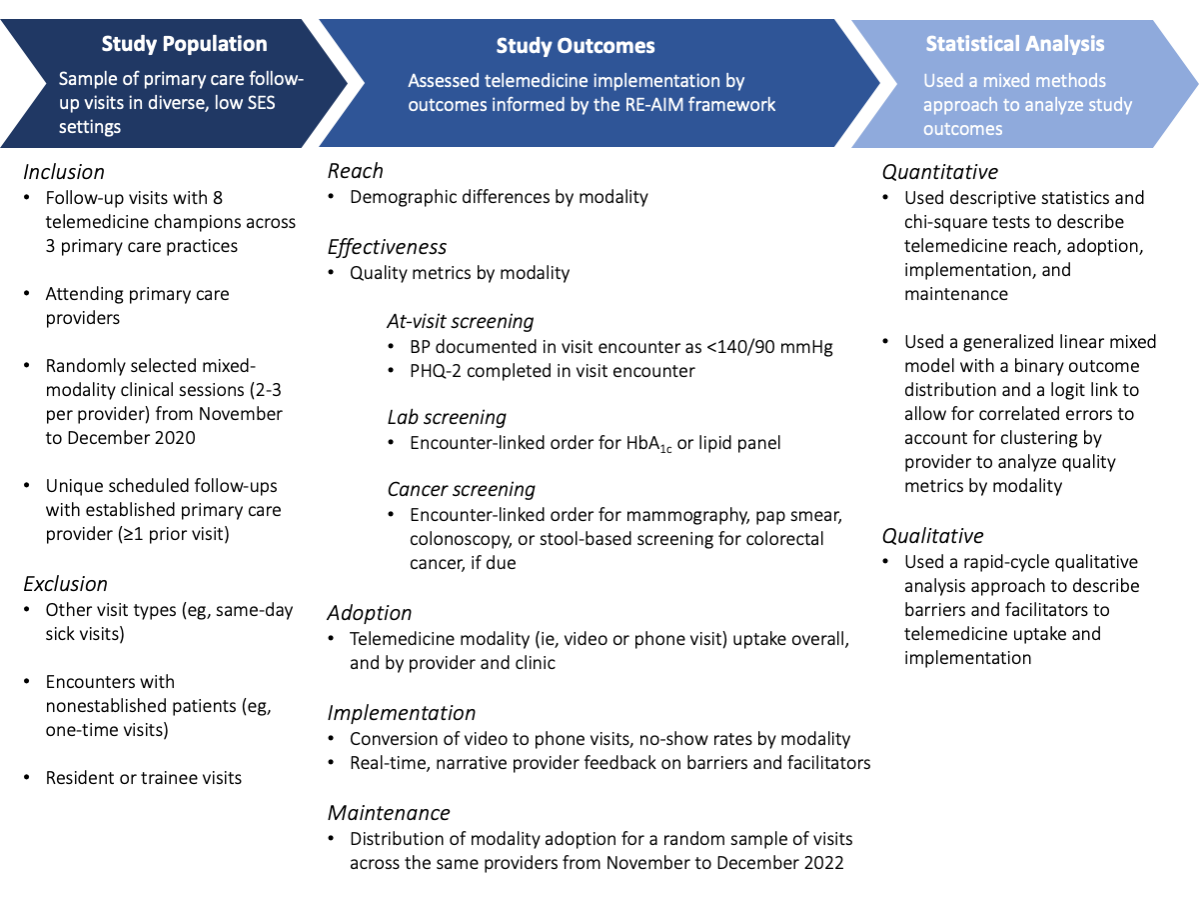
Study population, outcomes, and statistical analyses. Patients were determined to be due for cancer screenings based on United States Preventive Services Task Force recommendations. BP: blood pressure; HbA_1c_: hemoglobin A_1c_; PHQ-2: Patient Health Questionnaire-2; RE-AIM: Reach, Effectiveness, Adoption, Implementation, and Maintenance; SES: socioeconomic status.

From November to December 2020, we conducted a retrospective cohort study of unique patient visits among 8 attending telemedicine champions (to ensure adequate distribution of modalities) at 3 primary care practices in an academic medical center in New York City serving a predominantly (>80%) Medicaid population. We randomly selected 2-3 mixed-modality clinical sessions (ie, scheduled in-person, video, and phone visits) per provider. Included visits were follow-ups with established patients of attending primary care providers (PCPs); modality was determined by PCP recommendation or patient preference. We excluded other visit types (eg, same-day sick visits, new patient encounters, resident or trainee visits, and visits with nonestablished patients) to ensure that all visits were conducted by the patient’s PCP, allotted the same duration (~20 min), and used the same template in the electronic health record across modalities (Figure 1).

### Study Outcomes

Outcomes were informed by the RE-AIM (Reach, Effectiveness, Adoption, Implementation, and Maintenance) framework [16]. We assessed *reach* (demographic differences by modality) and *adoption* (telemedicine uptake at the clinic, provider, and patient levels). For *effectiveness,* we identified United States Preventive Services Task Force and Accountable Care Organization quality metrics (ie, screening for blood pressure [BP], with BP<140/90 mmHg; depression; hemoglobin A_1c_ [HbA_1c_]; lipids; mammography; cervical cancer; and colorectal cancer). We then conducted retrospective chart reviews to assess at-visit BP and depression screening as well as HbA_1c_, lipid, and cancer screening orders. For *implementation*, we described conversion of video to phone visits and no-show rates. Given the ongoing pandemic, we obtained provider feedback on barriers and facilitators using rapid-cycle qualitative research methods to reduce time commitment and intensity of data collection required of providers and to inform real-time practice changes [17,18]. Following clinical sessions, providers were emailed a web-based form to input free-text narrative comments on barriers to and facilitators for each encounter; the form was returned 1-7 days after the visit (Multimedia Appendix 1). For *maintenance*, we examined adoption rates in a random sample of visits identified using the same inclusion/exclusion criteria over an equivalent period, 2 years later (November-December 2022).

### Statistical Analysis

#### Quantitative Analysis

We used descriptive statistics and chi-square tests to describe demographic differences by modality, telemedicine uptake, conversion of video to phone visits, and no-show rates. To analyze the association between modality and quality metrics, we used a generalized linear mixed model with a binary outcome distribution and a logit link to allow for correlated errors to account for clustering by provider, adjusting for age, race, ethnicity, language (non-English vs English), Charlson Comorbidity Index [19] (range 0.0-12.0), and prior telemedicine (ie, video and phone) visits (0 vs ≥1).

#### Qualitative Analysis

Informed by prior rapid-cycle qualitative analysis approaches, we focused on specific research questions on implementation (ie, telemedicine barriers and facilitators) [20] and used the framework method to analyze free-text comments in Microsoft Excel [18,21,22]. Following an unstructured familiarization phase, initial codes were developed, and in a subsequent coding phase, final themes were identified, with relevant qualitative data tabulated by theme.

### Ethical Considerations

Ethics approval was obtained from the Columbia University Irving Medical Center Institutional Review Board (IRB-AAAR5570). A waiver for informed consent was granted given the retrospective nature of our study. All data were properly secured and stored in a manner compliant with HIPAA (Health Insurance Portability and Accountability Act) on an encrypted server.

## Results

We identified 281 unique visits. Mean participant age was 63.3 (SD 15.1) years; 71.5% (201/281) were Hispanic, 15.3% (43/281) were Black, and 61.9% (174/281) were non-English speaking. Mean Charlson Comorbidity Index was 3.39 (SD 2.46).

### Reach and Adoption

Overall, 24.6% (69/281) of the visits were in person, 25.3% (71/281) were through video, and 32.7% (92/281) through phone calls; 17.4% (49/281) were no-shows; 27.8% (78/281) of patients had no prior telemedicine visits. Patients aged ≥65 years (vs those aged <65 years) were more likely to have in-person (46/69, 67% vs 23/69, 33%) and phone visits (52/92, 57% vs 40/92, 43%) and less likely to have video (26/71, 37% vs 45/71, 63%) and no-show visits (22/49, 45% vs 27/49, 55%; *P*=.002). Non-English–speaking patients (vs English-speaking patients) were marginally more likely to have in-person (45/69, 65% vs 24/69, 35%), phone (63/92, 68% vs 29/92, 32%), and no-show visits (31/49, 63% vs 18/49, 37%); the proportion of video visits was almost similar between the two groups (35/71, 49% vs 36/71, 51%; *P*=.08). PCP was significantly associated with modality (*P*<.001). Clinic (*P*=.65), race (*P*=.39*)*, and ethnicity (*P*=.81) were not significantly associated with modality.

### Effectiveness

We found marginally significant differences in BP screening for video (15/71, 21%), phone (16/92, 17%), and in-person (39/69, 57%) visits (adjusted *P*=.06), with significantly lower odds for video (adjusted odds ratio [AOR] 0.21, 95% CI 0.09-0.50; *P*<.001) and phone (AOR 0.16, 95% CI 0.08-0.30; *P*<.001) versus in-person visits. Similar trends emerged for depression screening (24/71, 34%; 12/92, 13%; and 41/69, 59%, respectively; adjusted *P*=.06), for video (AOR 0.35, 95% CI 0.14-0.86; *P*=.02) and phone (AOR 0.10, 95% CI 0.04-0.26; *P*<.001) versus in-person visits. For HbA_1c_ or lipid screening orders, we found significantly lower odds for phone versus in-person visits only (AOR 0.46, 95% CI 0.24-0.88; *P=*.02). We found no significant difference by modality for a combined metric of any cancer screening order (ie, mammography, colorectal cancer, or cervical cancer; Table 1).

**Table 1:** Quality metrics by completed visit modality (n=232).

Visit modality	Blood pressure screening^a^			Depression screening^b^			HbA_1c_ or lipid screening^c^			Cancer screening^d^ (n=76)	
	AOR^e^ (95% CI)	*P* value	AOR (95% CI)		*P* value	AOR (95% CI)		*P* value	AOR (95% CI)		*P* value
Video vs in-person visit	0.21 (0.09-0.50)	<.001	0.35 (0.14-0.86)		.02	0.61 (0.27-1.36)		.23	0.74 (0.11-5.00)		.76
Phone vs in-person visit	0.16 (0.08-0.30)	<.001	0.10 (0.04-0.26)		<.001	0.46 (0.24-0.88)		.02	0.55 (0.13-2.41)		.43

^a^Blood pressure (BP) documented at visit, with BP<140/90 mmHg.

^b^Patient Health Questionnaire-2 completed at visit.

^c^Hemoglobin A_1c_ (HbA_1c_) or lipid panel ordered at visit.

^d^Mammography, colorectal cancer, or cervical cancer screening ordered at visit, if due.

^e^AOR: adjusted odds ratio; AOR is adjusted for age, race, ethnicity, primary language (non-English vs English), Charlson Comorbidity Index, prior telemedicine (ie, video and phone) visits (0 vs ≥1), and clustering by provider.

### Implementation

Overall, 39.4% (56/142) of scheduled video visits converted to phone visits. We found no significant difference in no-show rates by modality (*P*=.17). Emerging themes included technological issues, patient preferences, and need for social support. Interpreters or relatives serving as translators were often identified as facilitators for non-English–speaking patients versus English-speaking patients (Table 2).

**Table 2:** Provider-identified barriers and facilitators to telemedicine uptake.

Theme and category				Example quotes	
**Barriers**					
	Difficulty with technology setup		“Does not know how to set up apps on smartphone” “Low tech literacy” “Couldn’t figure out log in” “Patient is quite unfamiliar with technology, did not know what MyChart was…didn’t feel comfortable following the texted link to connect to Doximity video visit”		
	Lack of video-compatible device or technology		“Patient does not own a smart phone, no household contacts with a smart phone” “Only has landline” “Lack of Wi-Fi access”		
	Patient preference for in-person visits		“Really prefers in-person, feels like she gets better care” “Very resistant to video/phone visit, doesn’t believe getting adequate care”		
	Patient preference for phone visits		“Declined video, not comfortable with apps” “Patient requested phone visit”		
	Video platform issues (eg, Epic-MyChart, Zoom, or Doximity)		“Kept freezing when we tried Doximity and Epic/Zoom” “Could not figure out Zoom/MyChart (we tried on 3 different cell phones before we were successful)” “Doximity unsuccessful”		
	Poor connectivity		“Poor connection on cellular network” “Audio connectivity issues at the beginning of the call…video was a little grainy but still preferable to nothing.” “Patient had terrible connectivity due to Wi-Fi connection (he lives in a shelter), he could not get the audio to work”		
	Inadequate social support or setting		“Unstable social situation (living in a shelter with her two children)” “Lives alone without younger family members to help navigate tech-based platforms” “Limited privacy for video visit”		
**Facilitators**					
		Assistance from a relative			“Son and niece helped coordinate call” “Daughter had to patch patient in for a 3-way call” “Son and niece helped coordinate call, also used an interpreter” “Patient’s English-speaking daughter was next to her and helped her set-up”
		Technologic proficiency			“Very comfortable using technology” “Has smartphone, digitally savvy” “Patient is generally more tech-savvy…previously connected to MyChart for prior video visits both with me and with specialists; uses MyChart to check lab results and send messages”

### Maintenance

After 2 years, the distribution of in-person, video, and phone visits was 90.2% (111/123), 7.3% (9/123), and 2.4% (3/123), respectively.

## Discussion

### Principal Findings

After the first COVID-19 wave, we found similar distributions of in-person, video, and phone visits in low SES settings, with marginally better BP and depression screening for in-person care. De-adoption was marked within 2 years in our population.

Research delineating audio-only telemedicine and quality metrics is limited [5-7]. Our mixed methods findings highlight competing, disruptive technological barriers (ie, over one-third of video visits converted to audio-only visits) in low SES settings, potentially impacting quality metrics. We particularly observed gaps in screenings supported by ancillary clinic staff during in-person rooming (eg, medical assistants routinely perform BP and depression screenings), which was unavailable for telemedicine in our setting. Prior research shows an association between medical assistant–supported virtual rooming and successful video visit connections [23]; future studies should assess impact of virtual rooming on quality outcomes. Modality may be a proxy for other provider (eg, cognitive load, engagement, or shared decision-making) and patient factors (eg, transportation, access, or literacy), further impacting lab and cancer screening orders, which providers knew warranted travel. Some providers were more likely to use telemedicine, perhaps due to differences in preference, experience, or technologically savvy patient panels.

Our qualitative findings support prior qualitative research on determinants of telemedicine and remote patient monitoring, expanding on perspectives in historically marginalized populations [3,24,25]. Experts should examine whether addressing barriers (eg, technology access), promoting facilitators (eg, social support), and fine-tuning hybrid in-person and telemedicine models improve quality metrics and long-term telemedicine uptake. Future studies should assess within-modality differences in quality metrics by patient race, ethnicity, and language, given the documented disparities in telemedicine modality uptake. Special consideration is warranted for low-income settings, where in-person care equalizers may be unavailable (eg, video translator services, at-home labs, or device loan programs).

### Study Limitations

Limitations include unmeasured differences between modalities, including visit quality (eg, nonverbal communication and patient-provider rapport) and duration. Our sample size likely impacted cancer screening results, as few patients were due for screening. We were unable to assess provider or patient modality preferences (eg, providers less comfortable with video-based telemedicine scheduling more phone visits, older patients preferring in-person care, and technologically savvy patients using video visits), though we adjusted for clustering by provider, patient demographics, and prior telemedicine use. Additionally, telemedicine champions were used to ensure adequate distribution of modalities, which introduces bias; gaps are likely wider, overall. Lastly, we only describe 2 snapshots in time, albeit our results suggest telemedicine de-adoption in our population. Modality preferences, technological barriers, and restructuring of clinic workflows following the pandemic may have contributed to the reversion to in-person care and should be assessed as potential drivers of de-adoption.

### Conclusions

Proponents of widespread telemedicine implementation should consider telemedicine impact on quality outcomes, provider and patient preferences, and mitigating strategies in historically marginalized populations.

## Appendix

Multimedia Appendix 1 Web-based form for rapid-cycle qualitative feedback from providers on barriers to and facilitators for telemedicine uptake and implementation.

## Abbreviations

AOR: adjusted odds ratio
BP: blood pressure
HbA_1c_: hemoglobin A_1c_
HIPAA: Health Insurance Portability and Accountability Act
PCP: primary care provider
RE-AIM: Reach, Effectiveness, Adoption, Implementation, and Maintenance
SES: socioeconomic status

## Data Availability

The data sets generated and analyzed during this study are available from the corresponding author on reasonable request.
